# Effectiveness of memory bias modification in reducing depression and rumination symptoms and autobiographical memory bias: a pilot study

**DOI:** 10.3389/fpsyg.2023.1145259

**Published:** 2023-05-24

**Authors:** Haniyeh Sadat Atashipour, Fereshte Momeni, Behrooz Dolatshahi, Mahjube Sadat Mirnaseri

**Affiliations:** ^1^Department of Clinical Psychology, University of Social Welfare and Rehabilitation Sciences, Tehran, Iran; ^2^Faculty of Educational Sciences and Psychology, Shahid Beheshti University, Tehran, Iran

**Keywords:** memory bias, rumination, memory bias modification, cognitive biases, depression

## Abstract

There is a strong interest in cognitive bias modification as a new intervention that targets key underlying vulnerability factors of depression. Memory bias is believed to be a risk factor for the onset and maintenance of depression. In this study, we aimed to examine the effectiveness of memory bias modification on depression symptoms, ruminative thoughts, and autobiographical memory bias. We recruited 40 participants with mild depression who were randomly assigned to two groups of positive (*n* = 20) and neutral (*n* = 20) training. The participants were instructed to read and learn French-paired words with their Farsi translations. Next, they were encouraged to recall positive or neutral Farsi translations of French words according to their groups in the first session. After training, and in the second session (follow-up), they were asked to recall all the Farsi translations of the French words. Data were collected using Beck Depression Inventory II (BDI-II), Rumination Response Scale (RRS), Self-Referent Encoding Task (SRET). Analysis of covariance (ANCOVA) and logistic regression were used to analyze the data. Repeated retrieval practices resulted in better recall of the target words of the training in both conditions. Still, none of the groups had significant changes in depression scores, ruminative thoughts, and emotional aspects of memory bias. Our results suggest that two sessions of modifying memory biases were not sufficient for reducing the symptoms of depression and rumination. Implications of the finding from this study for future works are further discussed.

## 1. Introduction

Depressive disorders include a group of heterogeneous disorders that differ in terms of clinical appearance and intensity. Therefore, a variety of symptoms are recognized in these disorders and are regarded as one of the most common psychiatric disorders which are the leading cause of referral to health care centers (Sadock, 2015). Major depressive disorder (MDD) represents the classic condition in this group of disorders and is characterized by discrete episodes of at least 2 weeks duration involving clear-cut changes in affect, cognition, neurovegetative functions, and inter-episode remissions (American Psychiatric Association, 2013). It is common to have subclinical depressive episodes before the onset, which could be risk factors for the development of MDD (Horwath et al., 1992). Moreover, each depressive episode increases the likelihood that individuals will develop a subsequent episode of MDD (Solomon et al., 2000).

Cognitive theories emphasize the critical role of cognitive biases in information processing and the onset and maintenance of emotional disorders (Clark and McManus, 2002). Beck posited that cognitive biases influence the etiology of depressive episodes and argued that internal mental representations affect how depressed individuals perceive themselves and the world (LeMoult and Gotlib, 2019). Thereby, stressful life events activate negative thoughts and schemas, leading to congruent biases in attention, interpretation, and memory (Beck, 1979). Likewise, Ingram suggests that when negative appraisals of life events activate depressive memory networks, individuals tend to be more attentive to the information congruent with the triggered negative condition. As a result of the reprocessing of negative information in various memory networks, the detailed information is coded more deeply in depressive memory networks. This detailed biased memory increases the vulnerability to depressive disorders and can predict the next depression episode (Everaert et al., 2012). In depressive disorders, the most stable type of negative cognitive bias is memory bias (Matt et al., 1992; Gotlib and Krasnoperova, 1998; Gupta and Kar, 2012; Marchetti et al., 2018; Hitchcock et al., 2020), meaning that negative information is recalled better and more frequently than neutral or positive information (Gotlib and Joormann, 2010). This is especially true for self-relevant information such as self-descriptive adjectives (Matt et al., 1992; Symons and Johnson, 1997; Benau et al., 2019). For example, a negative self-relevant information bias in an individual may consist of describing her/himself as a loser but not a hard worker.

In addition to memory bias, one of the hypotheses about cognitive vulnerability to depression include the response styles theory (Nolen-Hoeksema, 1991). Rumination is one of the thought patterns that play an important role in depressive disorders. Depressive rumination as a verbal process is a response to distress and sadness which includes repetitive and passive concentration on distress symptoms, the possible causes, and the consequences of these symptoms (Nolen-Hoeksema et al., 2008). In depressive rumination, negative events are retrieved from memory and brought to mind repeatedly. This process facilitates future recalls of negative events and reinforces negative memory bias (Hertel, 2004; Hertel and Brozovich, 2010). Therefore, individuals suffering from depressive symptoms report more general and negative memories and information than their counterparts without depression (Williams et al., 2007).

In recent years, cognitive bias modification (CBM) is one of the digital therapeutic interventions and has been defined as the direct manipulation of automatic cognitive processes that are hypothesized to contribute to the development and maintenance of psychopathology. This manipulation is produced by extended exposure to task contingencies that favor predetermined patterns of processing selectivity (MacLeod and Mathews, 2012).

Cognitive bias modification is based on the relationship between cognitive biases and emotional symptoms. In essence, CBM is not designed to change how individuals respond to negative thoughts but rather to directly change the cognitive process that gives rise to such thinking (MacLeod and Mathews, 2012). This field includes three new therapeutic approaches: general training on memory, emotional memory, and attention and interpretation which affects memory processing more positively (Becker et al., 2015). Across all methods, a target cognitive bias is manipulated in which participants are taught to preferentially attend to process or otherwise engage with specific types of stimuli (i.e., positive or neutral), while simultaneously avoiding others (i.e., negative or threatening). In CBM-Memory, the repeated retrieval of target information facilitates the long-term retention of the learned material. Furthermore, positive memory schemata are repeatedly activated, which strengthens memory schema (Vrijsen et al., 2019). CBM interventions are appealing because of their accessibility and scalability, as they consist of brief sessions of a computer-based task, possibly administered online (Fodor et al., 2020).

Given the robust role of negative memory bias in depression (De Raedt and Koster, 2010; Gotlib and Joormann, 2010), CBM-Memory has emerged as a promising novel approach in cognitive treatments. For instance, autobiographical episodic memory-based training (AET) manipulates autobiographical processing in order to treat mood, anxiety, and stress-related disorders (Hitchcock et al., 2017). Hitchcock et al. (2017) reviewed 15 studies that compared an AET program to a control condition in samples with a clinician-made diagnosis. Results demonstrated promising evidence for the effectiveness of AET in the treatment of depression (*d* = 0.32). Arditte Hall et al. (2018) compared a positive to a neutral autobiographical memory training condition in a sample of 27 patients with depression to directly change the quality of positive autobiographical memory. In a series of trials, participants first recalled a sad memory in order to induce a negative mood state. Then, they recalled a happy memory and completed procedures to evoke a vivid, here-and-now quality of the memory. Training procedures were hypothesized to promote mood via the recall of increasingly vivid and specific positive memories. The results showed that positive training increased the specificity of autobiographical memory and mood regulation and, boosted the perceived ability to relive positive memories. Nevertheless, affective experience of training participants after the recall of positive memories did not differ from those of the control groups’ neutral memories. In another attempt to explore the effectiveness of CBM-Memory, Vrijsen et al. (2016) compared three study trials (positive, negative, and no-training). In this study, participants studied positive and negative word pairs (Swahili cues with Dutch translations; Vrijsen et al., 2016). The positive and negative conditions were followed by a cued-recall test of training-congruent translations; the no-training condition simply studied the pairs. Recall of the translations was tested after the training and after one week. Both recall tests revealed evidence of training-congruent bias which was also associated with emotional autobiographical memory. Additionally, the positive retrieval practice resulted in a stable positive mood, in contrast to the other conditions. The results suggested that memory retrieval practices based on CBM-memory can affect mood, and these training effects are transferred to autobiographical memory. Also, Vrijsen et al. (2019) compared positive to neutral CBM-Memory training in highly-ruminating and depressed individuals (Vrijsen et al., 2019). In both studies, participants studied positive, neutral, and negative Swahili words paired with their translations in five study–test blocks. The concluded Retrieval practice resulted in training-congruent recall both immediately and 1 week after the training, but there was no differential decrease in symptoms or autobiographical memory bias between the training conditions. Furthermore, in the dysphoric sample, positive training lead to a more positive bias of autobiographical memory in individuals who were previously positively biased. Respectively, Visser et al. (2020) developed a smartphone-based autobiographical memory training and showed that positive training increased positive memory bias significantly, while memory bias post-training did not significantly change in groups. In the same vein, Jopling et al. (2020) examined whether training individuals with depressive symptoms to remove negative information from working memory for 6 days would reduce depression symptoms and levels of rumination. Participants exhibited significant improvements from pre- to post-training in removing negative information from working memory, symptoms of depression, and rumination. In another study, Perlman et al. (2022) used a CBM procedure to train causal inferences and assess training effects on ruminative thinking, memory, and negative mood among people with different levels of depression. Training had immediate effects on negative mood and rumination but not after the recall of a negative autobiographical memory. In the most recent study, Bovy et al. (2022) used smartphone-based autobiographical memory training for 6 days to increase positive memory recall and thereby alter negative memory bias. A total of 96 dysphoric participants were allocated to three training conditions. Positive memory bias significantly increased from pre- to post-training after positive and sham interventions, suggesting an unspecific training effect. No transfer to memory specificity, implicit memory bias, and depressive symptoms was found. Also, the training effect was not influenced by the pre-existing level of positive memory bias. Following the initial COVID-19 Pandemic, a *post hoc* follow-up measurement revealed that subjects who benefitted most from either of the trainings were able to control their stress levels better during a natural stressful period, compared to those who responded poorer to the training.

On the grounds of moderate effectiveness of the CBM interventions on memory bias in depressive disorders (Vrijsen et al., 2016, 2019; Hitchcock et al., 2017; Arditte Hall et al., 2018; Jopling et al., 2020; Visser et al., 2020; Perlman et al., 2022), developing preventive protocols seems to be essential for averting or postponing future episodes of depressive disorders in vulnerable individuals. Nonetheless, only a few studies have examined CBM-Memory and further studies are needed to replicate results with different samples and populations. Thus, the current study aimed to examine whether positive training compared to neutral training will reduce depression and rumination symptoms and increase positive bias in autobiographical memory. We also expected that positive training would strengthen positive cognitive schemas by activating them repeatedly, which could result in a greater decrease in the recall of neutral and negative words.

## 2. Materials and methods

### 2.1. Participants

**Figure 1:**
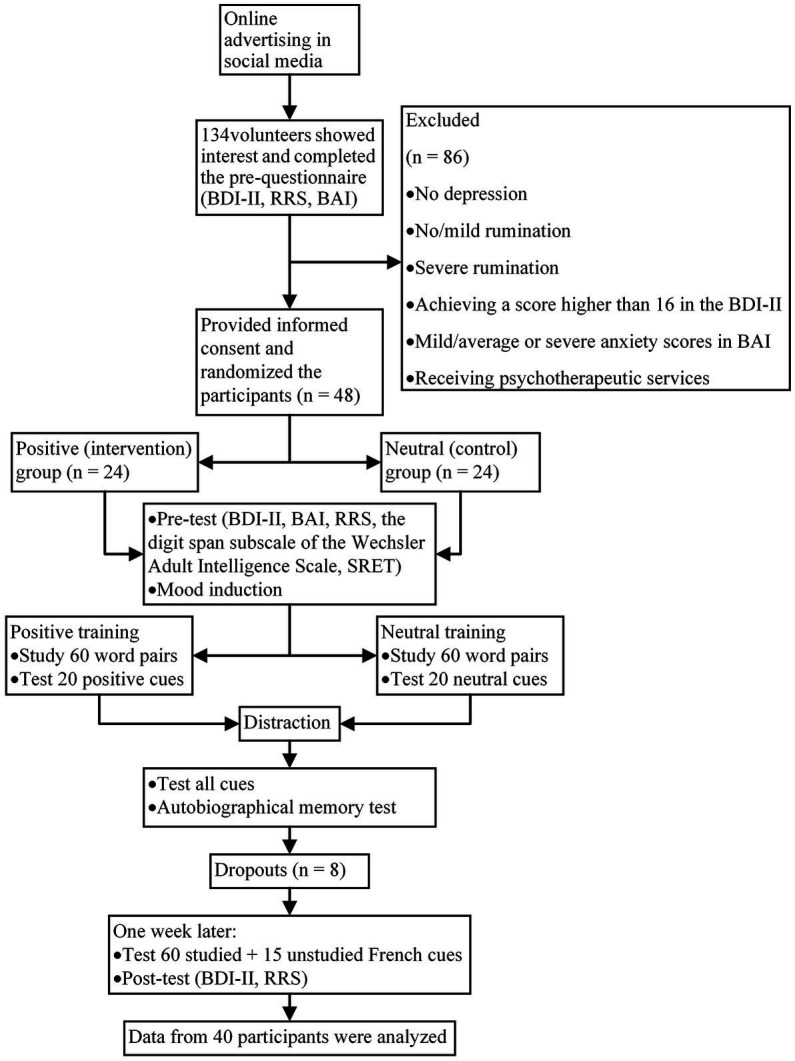
Process chart from recruitment to post-tests.

A quasi-experimental design with pretest-posttest was used. The participants were 40 university students aged 26–29 years old (*M*_age_ = 27.50, *SD* = 3.27; 72.5% females) recruited through online advertising in social media. One hundred and thirty-four individuals were interested in participating in the study and completed the pre-questionnaires from which a total of 52 individuals were considered for the initial interview based on exclusion/inclusion criteria (Table 1). According to Cohen’s sample size formula for experimental designs, considering *α* = 0.05 and *d* = 0.5, there is a statistical power of 88% with 20 participants per group (Cohen, 2013), though, considering the possibility of dropouts we randomly assigned 48 individuals to the two training groups of positive and neutral CBM-memory (24 per group). Our training included two sessions (one training session and a follow-up session) over 2 weeks. Eight participants dropped out before completing the second session due to a lack of cooperation and not responding to the Autobiographical Recall task. Therefore, the data of 40 participants from the training groups (20 per group) were analyzed. A graphic depiction of the recruitment process and training program is presented in Figure 1.

**Table 1:** Inclusion and exclusion criteria.

Inclusion criteria	Exclusion criteria
At least 18 years old.	Severe anxiety scores in Beck Anxiety Inventory (BAI).
Persian mother tongue.	Low scores on the digit span subscale of the Wechsler Adult Intelligence Scale (WAIS).
Having an education higher than a high school diploma.	Familiarity with the French language.
Having mild depression based on Beck Depression Inventory (BDI-II) scores (11–16).	Receiving any form of psychotherapeutic services.
Having moderate rumination based on Rumination Response Scale (RRS) scores (33–55).	

### 2.2. Procedure

This study was first reviewed and approved by the research ethics committee of the University of Social Welfare and Rehabilitation Sciences (Code Number = IR.USWR.REC.1398.094). Participants were informed about the aims of the study and the confidentiality of the data gathered. Then, they provided a signed consent form. Participants were randomly assigned to two training groups of positive (*n* = 24) and neutral (*n* = 24) CBM-memory. They were then administered the pre-assessment [BDI-II, Rumination Response Scale (RRS), Beck anxiety inventory (BAI), the digit span subscale of the Wechsler Adult Intelligence Scale, and Self-Referent Encoding Task (SRET)].

To design the Persian-adapted computer version of the SRET, in line with Vrijsen et al. (2019), 24 adjectives with depressive contents (12 positives and 12 negatives) were selected out of Affective Norms for 362 Persian Words (Bagheri et al., 2016). Affective words are rated according to their emotional valence, arousal, imageability, and familiarity in the Persian language for emotional word processing. To examine its validity, the task was given to an independent pilot sample (*n* = 16) along with the BDI-II. Correlation between the negative and positive bias scores and depression score was examined, and the results (*r* = 0.54, *p* = 0.030) indicated that depression score is significantly related to memory bias, supporting the validity of the Persian SRET.

Pre-existing memory bias, therefore, was assessed using the SRET. Before we started the training phases (first phase: study-test/second phase: test), a piece of Russian instrumental music was played to induce a negative mood (Vrijsen et al., 2019). Afterward, the participants received the CBM-memory in two phases of the study-test and test. In the study-test phase, participants were tested according to their groups (positive or neutral), but in the test phase, participants were tested on all words. After finishing both phases, by Autobiographical Recall Task, the participants were asked to speak about two influential events related to the past. A week later, during the follow-up session, the participants received only the test phase, and then they completed BDI-II and RRS as post-test.

### 2.3. Measures

#### 2.3.1. Beck depression inventory

Beck depression inventory II includes 21 items that have been designed for assessing the presence and intensity of depression symptoms. Each item consists of four sentences and is scored 0–3 on a Likert scale. The total score ranges from 0 to 63. A higher number in BDI-II indicates more severe depression. The internal consistency of the main version is high (*α* = 0.92, 0.93) and test–retest reliability (*r* = 0.93) is also reported. The Persian version of this questionnaire which has been normalized by Stefan-Dabson et al. (2007), is reported to have high internal consistency (*α* = 0.91; Stefan-Dabson et al., 2007).

#### 2.3.2. Rumination response scale

Rumination response scale includes 22 items that have been designed for assessing rumination and the tendency toward rumination as a response to depressive mood (Nolen-Hoeksema, 1991). Each item consists of four sentences and is scored 1–4 on a Likert scale. The total score ranges from 22 to 88. A higher number in RRS indicates more severe rumination. The internal consistency of the main version is high (Cronbach’s alpha varied from 0.88 to 0.92) and test–retest reliability (*r* = 0.67) is also reported. Internal consistency of the Persian version is computed for the Iranian population (*α* = 0.88; Bagherinezhad et al., 2010).

#### 2.3.3. Beck anxiety inventory

Beck anxiety inventory includes 21 items that have been designed for assessing the severity of a range of anxiety symptoms. Each item consists of four sentences and is scored 0–3 on a Likert scale. The total score ranges from 0 to 63. A higher number in BAI indicates more severe anxiety. Beck et al. (1988) calculated the internal consistency (*α* = 0.92) and test–retest reliability (*r* = 0.75) of this questionnaire. The Persian version of this questionnaire which was validated by Kazemi (2003), yielded acceptable internal consistency (*α* = 0.78).

#### 2.3.4. The digit span subscale of the wechsler adult intelligence scale

This scale is short-term memory and attention test. The participants are expected to recall and repeat the auditory information, respectively, (Marnath, 2016). This subscale is applied separately in two forward and backward spans. Each section is scored from 0 to 14 and to calculate the total score of this subscale, the obtained scores of both tests are added up. The highest possible score for this subscale is 28.

#### 2.3.5. Self-referent encoding task

The computerized Self-Referent Encoding Task (SRET) is used to assess self-referent negative memory bias (Derry and Kuiper, 1981; Dobson and Shaw, 1987). This is an implicit learning task that consists of an encoding phase followed by a recall phase. During the encoding phase, 12 positive and 12 negative possibly self-descriptive adjectives are individually presented on a computer screen in a fixed randomized order. These words are aimed at triggering positive and negative cognitive schemas (Beck, 1987; Young, 1999). In this study, the participants were asked to focus on the middle of a computer screen and determine if the word arising describes them or not. After finishing the task and 2 min of distraction through doing a math calculation (i.e., successive subtraction of 7 from 100), a piece of paper was given to the participant, and they were asked to write down the words they recalled from the first task. To calculate the score of positive memory bias, the self-describing positive words and the recalled positive words of the task are divided by the total number of positive and negative words that were considered to be self-describing (whether recorded or recalled). The same is applied to calculating negative memory bias scores (Vrijsen et al., 2019).

#### 2.3.6. Autobiographical recall task

Following training, we asked for autobiographical recall of two events. First, we asked participants to recall and then type the description of a personal event from the day before that made an impression on them, and to identify the corresponding feeling. Second, we asked them to type a description of an important life event. Here, they were told that it could be something that happened recently or something that happened in the distant past, as long as it evoked a feeling from them (Vrijsen et al., 2019). Three independent raters blinded to the participants’ training conditions scored these personal events as positive or negative.

### 2.4. Intervention

To design the Persian-adapted training program for CBM-memory based on Vrijsen et al. (2019), 25 positive, 25 negative, and 25 neutral words were selected from the word collection of Bagheri et al. (2016). Considering the idea of training which was matching individuals’ native language as meaningful emotional target words with a language that individuals do not know and its words are without meaning to participants, we required an unfamiliar language for the word pairs. Thereby, the Persian words were translated into French. The training included two phases of study-test and test. The study-test phase included showing 60 paired words to the participants and asking them to memorize the paired words. After doing a math calculation for inducing distraction, 20 positive French words and 20 neutral French words were shown to the participants in positive and neutral groups, respectively. The participants were asked to type the Persian translation of each French word. The words would appear one by one on the monitor with fixed timing. This two-step of study-test phase was repeated five times for each participant. After the end of the study phase and 4 min of break time, the test phase started with 60 words appearing on the screen in the same order as the study phase, while the participants were asked to type the Persian translation of each word. A week later during the second session, the participants received only the test phase including 75 French words, 15 of which were new (five negatives, five positive, and five neutral words). These new words would appear between the 60 old words to assess the general bias and the false memory of the participants.

### 2.5. Data analyses

We used SPSS 23 software for data entry and statistical analyses. The normality of the distribution for outcome measures was tested using the K-S test and the results supported the normality of the data. The chi-square was used to detect initial differences between groups in age, marital status, and level of education. We performed a series of analyses of covariance (ANCOVA) to determine whether the groups differed in their reported levels of rumination and depression symptoms and reported emotional aspects of memory bias using baseline values, anxiety, and short-term memory as covariates. Of note, given the high comorbidity between depressive and anxiety disorders, the anxiety score of the participants was considered as a covariate to control its influence on the outcome variable. Likewise, as individual differences in memory ability could affect our result, we controlled the short-term memory ability variable. The following rules of thumb are used to interpret values for Partial eta squared*: η_p_^2^* = 0.01 indicates a small effect; *η_p_^2^* = 0.06 indicates a medium effect; *η_p_*^2^ = 0.14 indicates a large effect. It was decided beforehand that a *p level* of <0.05 would be accepted as indicating statistically significant results. To investigate whether the training effects transfer to memory processes, we used separate logistic regression models for recent and lifetime autobiographical memory. Both models included the predictors of practice condition, baseline bias (positive vs. negative memory), and the interaction between these variables to predict the valence (positive vs. negative) of autobiographical memory. The significance of logistic coefficients was tested using the Wald test. The level of statistical significance was considered at 5%.

## 3. Results

**Figure 2:**
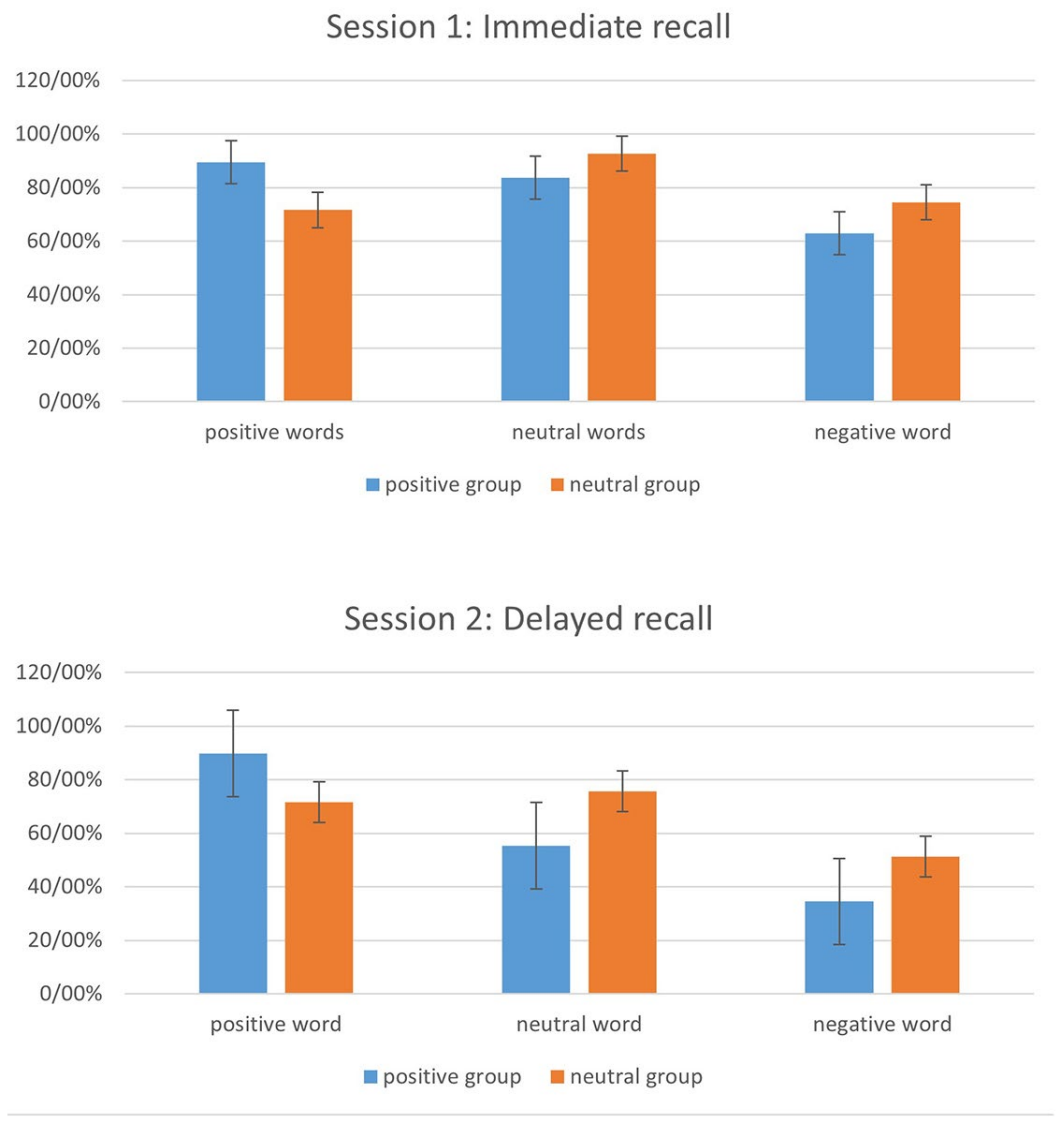
Percentages of translations recalled on the immediate and the delayed test. Error bars represent standard deviation (SD).

As shown in Table 2, groups did not differ significantly on demographic variables, indicating that the groups were matched in these variables. Also, descriptive information on study variables is presented in Table 3. The results of the ANCOVA analysis indicated that controlling the baseline scores, memory, and anxiety scores, no significant difference was found in the post-test scores of depression [*F*(1, 34) = 0.74, *p =* 0.390*, η_p_^2^* = 0.020] and rumination [*F* (1, 34) = 0.15, *p =* 0.700, *η_p_^2^* = 0.780] between the two groups. Also, ANCOVA was used to investigate whether the training make a significant difference between the two groups in translations recalled on post-tests. Pre-tests, memory, and anxiety scores were applied as covariate variables, and results showed that there is a significant difference between the two groups in recalled positive words on the immediate test [*F*(1, 34) = 20.463, *p =* 0.000, *η_p_^2^* = 0.376]. However, recall of negative and neutral did not show any difference between the groups. The results of the Bonferroni *post hoc* test show that the difference between the mean of the positive group in the recalled positive words in the first session was significant (*M* = 4.070, *SD* = 0900, *p* = 0.000). As well, there was a significant difference between the two groups in recalled positive [*F*(1, 34) = 6.057, *p =* 0.019, *η_p_^2^* = 0.151], negative [*F*(1, 34) = 6.094, *p =* 0.019, *η_p_^2^* = 0.152], and neutral [*F*(1, 34) = 6.763, *p =* 0.014, *η_p_^2^* = 0.715] words on delayed test in the second session. The results of the Bonferroni *post hoc* test show that the difference between the mean of the positive group in the recalled positive words was 2.881 (*M* = 2.881, *SD* = 1.170, *p* = 0.019) scores higher than the neutral group and the difference between the mean of the neutral group in the recalled neutral words was 2.821 (*M* = 2.821*, SD* = 1.085, *p* = 0.014) scores higher than the positive group. The neutral group also performed better in recalling negative words and showed a difference of 3.002 (*M* = 3.002, *SD* = 1.216, *p* = 0.019) scores from the positive group. Figure 2 illustrates the percentages of translations recalled on the immediate and the delayed test in groups.

**Table 2:** Descriptive information of demographic data in groups.

Variables	Groups		Comparison	
	Positive (*n* = 20)	Neutral (*n* = 20)	$x^{2}$	*p*
Marital status (%)				
Married	12 (60%)	11 (55%)	0.102	0.50
Single	8 (40%)	9 (45%)		
Education (%)				
Bachelor	10 (50%)	9 (45%)	0.105	0.95
Master	9 (45%)	10 (50%)		
Doctoral	1 (5%)	1 (5%)		
Age				
Female	26.86 (3/99)	26.87 (3/50)	7.73 ^a^	0.81
Male	27.33 (2/65)	28.80 (2/95)		

^a^Independent *t*-test was used to examine gender differences in age.

**Table 3:** Descriptive information of research variables.

Training conditions	Variables	Mean (*SD*)	Frequency (Percentage)
Positive	Pre-test BDI-II	13.15 (2.13)	
	Post-test BDI-II	12.35 (3.08)	
	Pre-test RRS	43.90 (8.15)	
	Post-test RRS	42.50 (7.38)	
	BAI	7.50 (4.42)	
	WAIS	12.15 (2.32)	
	Pre-positive memory bias	0.60 (0.08)	
	Post-positive memory bias (recent)		12 (60%)
	Post-positive memory bias (lifetime)		10 (50%)
Neutral	Pre-test BDI-II	13.05 (2.13)	
	Post-test BDI-II	13.05 (4.19)	
	Pre-test RRS	46.05 (10.45)	
	Post-test RRS	45.00 (11.34)	
	BAI	7.25 (3.74)	
	WAIS	13.15 (2.91)	
	Pre-positive memory bias	0.59 (0.11)	
	Post-positive memory bias (recent)		10 (50%)
	Post-positive memory bias (lifetime)		11 (55%)

SD, standard deviation; BDI-II, beck depression inventory; RRS, rumination response scale; BAI, beck anxiety inventory; WAIS, the digit span subscale of the Wechsler Adult Intelligence Scale.

Separate logistic regression models for both recent and lifetime event descriptions were conducted. Neither the main effect of the condition [recent: (*OR* = 1.500, 95% CI [0.429, 5.248]), lifetime: (*OR* = 0.818, 95% CI [0.236, 2.835])], the main effect of baseline bias [recent: (*OR* = 0.333, 95% CI [0.58, 1.907]), lifetime: (*OR* = 4.385, 95% CI [0.763, 25.204])] nor the interaction between condition and baseline bias was significant [recent: (*OR* = 1.088, 95% CI [0.305, 3.885]), lifetime: (*OR* = 1.286, 95% CI [0.361, 4.584])]. Therefore, memory bias modification did not increase the likelihood of experiencing positive autobiographical memory bias.

## 4. Discussion

In this study, we aimed to examine whether two sessions of CBM-Memory led to a more positive autobiographical memory bias, reductions in depression symptoms, and ruminative thoughts. The results showed that compared to neutral training, positive CBM-memory training did not reduce depression or rumination symptoms significantly, nor stimulated positive autobiographical recall in individuals with mild depression. However, consistent with previous studies (Vrijsen et al., 2016, 2019; Hertel et al., 2017), the retrieval training resulted in the training-congruent recall of emotional information in both conditions.

According to Beck’s theory of depression, we expected the participants in the positive group to recall positive target words because this may lead to strengthening their positive cognitive schemas relative to their negative schemas (similar to the positive daily keeping technique in CBT), and strengthening these positive schemas should protect against future schematic re-activation of negative bias and depression.

The results of this research are in line with some studies on depression but in comparison with rumination which had begun to develop training protocols for CBM-memory (Hertel et al., 2017; Vrijsen et al., 2019; Visser et al., 2020). Although the effect size of these studies should also be considered, the lack of effectiveness in mentioned studies in reducing depression symptoms and the current research on rumination symptoms might be explained by the following reasons. First, depressed individuals with negative memory bias and high trait-ruminating may need more professional help and practice in reducing this negative cognitive processing style. Second, these people also may have biases in describing their symptoms and have limited insight into their symptom change, and/or cognitive deficits that compromise self-monitoring of improvement (Corruble et al., 1999; Rush et al., 2006). Third, content-based models suggest that depressive thought is more negative for self-relevant than for externally-focused content. Process-based models propose that early, automatic processes are not negatively biased in depression, but that deeper processes are biased (Wisco, 2009). Forth, our training groups were positive and neutral and it could be less significant compared to having a negative training condition because neutral training is a lower dose of positive training. Moreover, we concluded that the sole use of CBM-Memory training for two sessions does not significantly increase the likelihood of positive bias in recalling recent and lifetime events. In a project by Vrijsen et al. (2016) for discovering the clinical usage of CBM-Memory, a training design was developed to study the model of the repeated retrieval of emotional data. This frequent retrieval forms an important aspect of depressive rumination. It was concluded that memory biased retrieval can affect mood, and training protocols can affect episodic memory (Vrijsen et al., 2016). Since the daily and repeated recall of desirable and positive experiences is known as an adaptive emotion regulation technique in individuals with negative affect and mood, the non-significant effectiveness of CBM-Memory could be explained by the fact that this recalling practice has only been done once a week (Erber and Erber, 1994; Rusting and DeHart, 2000). Another reason to explain not increasing positive bias in recalling recent and lifetime events may be related to the way the question is asked about the recent and lifetime events in Persian as the semantic load that is induced to the participant through the selected word can be effective in the directional recall of a negative or positive event. For example, using the two words “event” or “incident” could evoke a negative recall, while using the word “memory” may evoke an event as more positive and enjoyable. Finally, training in repeated retrieval of positive words led to better learning and remembrance of positive words compared to neutral and negative words, and training in the retrieval of neutral words led to better learning and remembrance of neutral words compared to positive and negative words. Considering the role of memory processes (repeat and review, expansion, and organization) in transferring the sensory stimuli received from short-term memory to long-term memory and facilitation of complete learning, in the current study, emotional target words were used to repeat and review (during five stages of study-test and test) and this led to better learning and recalling of the target words compared to the other words. These findings are in line with previous studies (Vrijsen et al., 2016; Visser et al., 2020) and, it could be stated that training for retrieval of emotional words, facilitates learning and recalling the target emotional words compared to other words. Our results can help future studies design CBM-Memory protocols with more sessions by concentrating on self-relevant content.

There were some limitations in this study. First, we had no information about how participants “memorized” word pairs. We tried to control the memory by using wechsler adult intelligence scale (WAIS), but we do not know which specific memory processes are affected by the training in everyone. Second, according to our results and the causal link between memory bias and depression, it is important to improve such treatments for depression that target the memory process and cognitive bias with more sessions in further research. Future research could aim to examine whether a positive CBM-Memory with more sessions and follow-up considering adding a control or sham group to training groups may yield stronger effects on depressive and rumination symptoms and autobiographical memory bias in vulnerable individuals. Third, as using self-rated depressive symptom measures are more conservative and less sensitive to change (Cuijpers et al., 2010), future research could utilize both self-rated and clinician-rated instruments for depression. Also, researchers can select self-relevant words in training CBM-memory (as we used some self-relevant stimuli words in SRET) for individualized material, which may result in stronger training effects as bias is especially strong for self-relevant information. Therefore, in line with content-based models, it would be better if future studies use self-relevant words such as “happy” instead of externally-focused content words like “happiness.” Also, future studies could examine the effects of this intervention on depression along with other common treatments for improving therapeutic outcomes. Finally, given the mentioned limitations, the result should be generalized cautiously and more studies are required in this field, especially in Iran.

## 5. Conclusion

The results of this study showed that regardless of yielding better recall of training congruent words, the two-session CBM-Memory protocol does not result in its significant transference to autobiographical memory bias, depression, and rumination symptoms.

## Data availability statement

The raw data supporting the conclusions of this article will be made available by the authors, without undue reservation.

## Ethics statement

The studies involving human participants were reviewed and approved by the research ethics committee of the University of Social Welfare and Rehabilitation Sciences, Tehran, Iran (IR.USWR.REC.1398.094). The patients/participants provided their written informed consent to participate in this study.

## Author contributions

HSA performed the intervention and prepared the manuscript. MSM analyzed data. FM and BD supervised the study and reviewed and revised the manuscript. All authors contributed to the article and approved the submitted version.

## Conflict of interest

The authors declare that the research was conducted in the absence of any commercial or financial relationships that could be construed as a potential conflict of interest.

## Publisher’s note

All claims expressed in this article are solely those of the authors and do not necessarily represent those of their affiliated organizations, or those of the publisher, the editors and the reviewers. Any product that may be evaluated in this article, or claim that may be made by its manufacturer, is not guaranteed or endorsed by the publisher.
